# Phylogenetic appraisal of antagonistic, slow growing actinomycetes isolated from hypersaline inland solar salterns at Sambhar salt Lake, India

**DOI:** 10.3389/fmicb.2013.00190

**Published:** 2013-07-10

**Authors:** Polpass Arul Jose, Solomon Robinson David Jebakumar

**Affiliations:** Department of Molecular Microbiology, School of Biotechnology, Madurai Kamaraj UniversityMadurai, India

**Keywords:** solar saltern, rare actinomycetes, ARDRA, phylogeny

## Abstract

Inland solar salterns established in the vicinity of Sambhar Lake are extreme saline environments with high salinity and alkalinity. In view of the fact that microbes inhabiting such extreme saline environments flourish the contemporary bioprospecting, it was aimed to selectively isolate slow growing and rare actinomycetes from the unexplored solar salterns. A total of 14 slow growing actinomycetes were selectively isolated from three composite soil samples of inland solar salterns. Among the isolates, four groups were formed according to similarity of the banding patterns obtained by amplified ribosomal DNA restriction analysis (ARDRA). A subset of representative isolates for each ARDRA group was identified using 16S rDNA sequence based phylogenetic analysis and subsequently the entire isolates were assigned under three different genera; *Streptomyces, Pseudonocardia*, and *Actinoalloteichus*. The genus *Streptomyces* was found to be the dominant among the isolates. Furthermore, rare actinomycete genus *Actinoalloteichus* was isolated for the first time from solar saltern. Determination of salt-tolerance revealed that certain level of salt-tolerance and moderate halophilism occurs among the actinomycetes isolated from the inland salterns. In addition, all the acinomycetes were screened in two levels to unravel their ability to produce antimicrobial compounds. Significant antimicrobial activity was found among the actinomycetes against a range of bacteria and fungi to worth further characterization of these persuasive actinomycetes and their antimicrobial secondary metabolites. In a nutshell, this study offered a first interesting insight on occurrence of antagonistic rare actinomycetes and streptomycetes in inland solar salterns associated with Sambhar salt Lake.

## Introduction

Actinomycetes are the source of most economically and biotechnologically important antimicrobial compounds (Mahajan and Balachandran, 2012). Exploring the members of the order Actinomycetales from various habitats have been continued to thrive with discovery of novel classes of bioactive compounds (Lam, 2006; Fu et al., 2011; Xu and Li, 2012; Tian et al., 2013). However, the exponential discovery of novel actinomycetes from conventional environments has significantly decreased in recent years (Zotchev, 2012). Identifying new sources of actinomycetes is a significant approach among the contemporary strategies deal with current need for new antibiotics (Poulsen et al., 2011). Hence, it is indispensible to focus on unexplored unique environments which could have evolved differently from that had already been analysed.

Hypersaline environments are extreme habitates, their actinomycete inhabitants are largely unexplored for discovery of novel bioactive secondary metabolites (Hamedi et al., 2013). In fact, several strides have been made to isolate actinomycetes from saline environments and most of them turn out to have potential biotechnological applications (Gulder and Moore, 2010; Vijayakumar et al., 2012; Hamedi et al., 2013). Solar salterns are unique hypersaline environments, characterized by their high salt concentration and alkaline pH (Zafrilla et al., 2010). In India, there are many coastal and inland solar salterns from which salt is produced for human consumption and industrial needs. Several actinomycetes, found to be proficient to produce antibiotic compounds and halotolerant enzymes, have been reported from the coastal solar salterns (Vasavada et al., 2006; Thumar and Singh, 2009; Thumar et al., 2010; Jose et al., 2011). More recently, diverse actinomycetes have been isolated and there phylogenetic diversity was reported from an Indian coastal solar saltern (Jose and Jebakumar, 2012). However, the inland solar salterns located in this country remain poorly described to this day.

The Sambhar Lake is the largest inland saline and alkaline Lake of India and covers an area of 230 sq km, situated in the middle of a closed depression in the Aravalli schists in Rajasthan (Upasani, 2008). In the east of the lake, there are solar salterns ponds where salt has been produced for a thousand years and made Rajasthan the third largest salt producing state in India. It produces 196,000 tons of salt every year, which equals 8.7% of India's salt production (Jain, 2005). Previous microbial studies on Sambhar Lake environment have revealed the occurrence of halophilic archeal species belonging to genera *Natronobacterium* (Upasani and Desai, 1990) and *Natrialba* (Upasani, 2008). Further, report of eubacterial diversity from this site is scarce with an isolation of haliphilic bacterial genus *Ectothiorhodospira*. With this background, the current work was aimed to selectively isolate and phylogenetically identify the actinomycetes from the inland solar salterns.

## Materials and methods

### Sample collection and physico-chemical analysis

**Figure 1:**
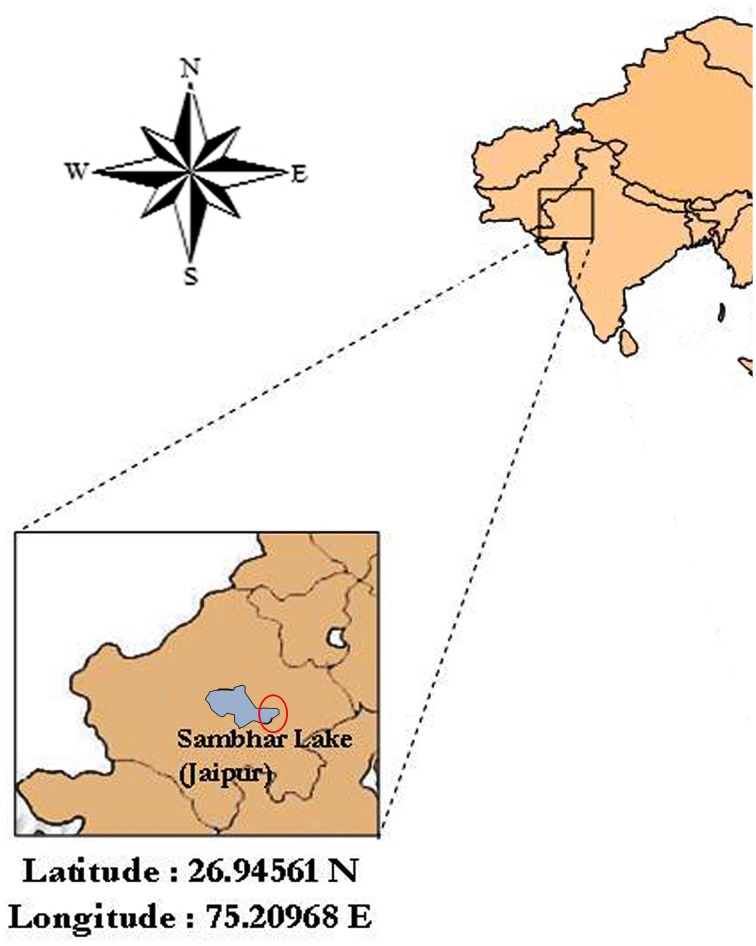
**Map of the location of the sampling site, Sambhar Salt Lake (about Latitude 26.94561 N and Longitude 75.20968 E), Rajasthan, India**. Fairly accurate location of sampling is encircled in the figure.

Ten soil samples were collected aseptically from crystallizer ponds of inland solar saltern located eastern side of the Sambhar salt lake (Latitude 26°58′N and Longitude 75°05′E), Jaipur, India (Figure 1). Among them, a soil sample was subjected to physiochemical analysis following standard methods to explore the physiochemical characteristics of the collection site. Soil pH, electrical conductivity (EC) and total nitrogen were determined according to Jackson (1973). Organic matter (organic carbon) was determined according to the method of Walkley and Black (1934). Total sodium, potassium and magnesium were estimated using atomic absorption spectrophotometer (AA-6200, Shimadzu, Japan).

### Isolation of actinomycetes

Three composite samples were prepared from the collected soil samples. The composite samples were homogenized and air-dried in a laminar flow hood, ground lightly with a sterile pestle, heated for 2 h at 60–65°C and finally stamped onto the isolation agar media using sterile cotton plug as described earlier (Gontang et al., 2007; Jose and Jebakumar, 2012). In another method, 1 g of composite soil sample was suspended in 4 ml sterile water containing 1% NaCl, heated for 6 min at 55°C, vigorously shaken and further diluted (1:4) in sterile water, and 50 μl of each dilution was spread with a sterile glass rod onto agar-based isolation media. The isolation media (IM) contained following components: IMa, 10 g of starch, 4 g of yeast extract, 2 g of peptone, 10 g of NaCl, 18 g of agar, and 1 l of distilled water; IMb, 10 g of starch, 4 g of yeast extract, 20 g of NaCl, 2 g of NH_4_SO_4_, 1 g of MgSO_4_.7H_2_O, 1 g of K_2_HPO_4_, 22 g of agar, and 1 l of distilled water; and IMc, 10 g of starch, 0.3 g of casein, 2 g of KNO_3_, 4.6 g of sodium chloride, 2 g of K_2_HPO_4_, 0.05 g of MgSO_4_.7H_2_O, 0.02 g of CaCO_3_, 0.01 g of FeSO_4_.7H_2_O, 1 mg of ZnSO_4_.7H_2_O, 18 g of agar and 1 l of distilled water. The isolation media amended with filter-sterilized cycloheximide (100 μg/ml) and nalidixic acid (5 μg/ml) to suppress the growth of gram-negative bacteria and fungi to favor the selective isolation of actinomycetes. The isolation plates were incubated at 30°C for 2–12 weeks to acquire slow growing and rare actinomycetes. Actinomycete like leathery colonies appeared after 2 weeks of incubation were selected and repeatedly streaked on IMb to get the pure isolates. The pure actinomycete like isolates were maintained either on IMb or trypticase soy agar (Himedia, India) slants supplemented with 1% of NaCl (w/v); for long-term storage, cultures were maintained in 18% glycerol at −20°C.

### 16S rDNA amplification

Isolates were grown in trypticase soy broth supplemented with 1% NaCl for 10 days and the genomic DNA of each isolate was extracted following standard phenol-chloroform extraction procedure (Hopwood et al., 1985). The 16S rDNA was amplified from genomic DNA obtained from the actinomycete isolates by PCR with eubacterial universal primer pair 27F 5′-AGAGTT TGA TCC TGG CTC AG-3′ and 1492R 5′-GGT TAC CTT GTT ACG ACT T-3′ (Lane, 1991). The reaction mixture contained 25 ng of DNA as template, 1X reaction Buffer (10 mM Tris pH 8.3, 50 mM KCl, 1.5 mM MgCl_2_), 200 μM of each dNTP, 10 pM of each primer and 0.05 U of Taq DNA polymerase (Sigma, USA). PCR conditions consisted of an initial denaturation at 94°C for 5 min; 31 cycles at 95°C for 30 s, 54°C for 90 s, and 72°C for 120 s; and a final extension at 72°C for 5 min. The amplification reactions were performed in Bio-Rad thermal cycler (MyCycler, Bio-Rad, USA) and the amplification products were examined by 1% agarose gel electrophoresis.

### Amplified ribosomal DNA restriction analysis (ARDRA)

The 16S rDNA amplification products were purified by using PCR product purification spin kit (HiPurA, HiMedia, India) following the protocol suggested by the manufacturer. To identify the number of polymorphic groups and select the representative strains among the actinomycete isolates, aliquots of purified 16S rDNA amplicons were subjected to amplified ribosomal DNA restriction analysis with *Hae*III and *Hinf*I following the previously described procedure (Jose and Jebakumar, 2012). The digestion reactions were carried out in 20 μL reaction mixture containing 1X recommended buffer, 1 μL (10U) of restriction enzymes and 10 μL of 16S rDNA amplicons at 37°C for 3 h. The digested restriction fragments were electrophoresed in 2.5 % agarose gel using TE buffer. The gel was stained with ethidium bromide and visualized under UV transilluminator. Strong and clear bands were scored in a binary data form and used for similarity and clustering analysis in numerical taxonomy analysis program package, NTSYS-pc 2.02i (Rohlf, 1998). Similarities among the isolates were calculated by Jaccard's coefficient (Jaccard, 1912) and the dendrogram was constructed using UPGMA method (Nei and Li, 1979).

### 16S rDNA sequencing and phylogenetic analysis

The PCR amplicons of 5 actinomycete isolates with representative ARDRA profiles were sequenced by Applied Biosystems 3730XL DNA Analyzer using same primer set as used in PCR amplification. All the sequences obtained from sequencing were analyzed and edited by using BioEdit software (Hall, 1999). Initially all the 16S rDNA sequences were compared to sequences in GenBank by use of the Basic Local Alignment Search Tool online service (Altschul et al., 1990) to determine approximate phylogenetic position. Sequences then were aligned using ClustalX (Thompson et al., 1997) with related 16S rDNA sequences retrieved from GenBank. Neighbor-joining phylogenetic tree was inferred from the 16S rDNA sequences of 5 representative isolates and selected members of related genus within the order Actinomycetales by using suitable programs of the PHYLIP (phylogeny inference package) version 3.68 (Felsenstein, 2008). The robustness of tree topology was assessed by bootstrap analysis of the neighbor-joining data sets on 1000 resamplings using the same PHYLIP package. Manipulation and tree editing were done by using TreeView.

### Morphology of representative strains

All the representative isolates obtained from ARDRA analysis were subjected to morphological characterization on IMb. Inoculated plates were incubated at 29°C for 12 days and their morphology was visually observed.

### Determination of salt tolerance

Salt tolerance of the actinomycete isolates was determined on starch nitrate medium prepared with series of NaCl concentrations; 0, 50, 70, 100, 150, 180, 200, 250, and 300 g/L (~0–5 M) following Kushner (1978). Dry weights of mycelial pellets were quantified by drying at 60°C as a measure of growth on different salt concentrations.

### *In vitro* screening for antimicrobial activity

Antimicrobial profile of all the actinomycte isolates was recorded against a range of bacterial and fungal strains using primary and secondary screening methods. Bacterial test strains viz., *Bacillus subtilis* MTCC 441, *Klebsiella pneumoniae* MTCC 109, *Salmonella typhi* MTCC 733, *Proteus vulgaris* MTCC 426 and *Staphylococcus aureus* MTCC 3160 were obtained from the IMTECH, Chandigarh, India. Fungal test strains viz., *Fusarium oxysporum, Aspergillus nigre*, and *Alternaria alternata* were obtained from Tamilnadu agricultural college and research centre, Madurai, India. The bacterial cultures were maintained either in Mueller Hinton broth (MH broth) or in nutrient broth. The fungal cultures were maintained in potato dextrose agar.

### Primary screening for antimicrobial activity

All the actinomycete isolates were primarily screened for antibacterial and antifungal activity against the test microorganisms using agar plug method (Eccleston et al., 2008) and dual culture method (Harveson and Kimbrough, 2000), respectively.

The actinomycetes were initially streaked over three different Production Media (PM) solidified with 2% agar: PM1, 10 g of starch, 4 g of yeast extract, 5 g of NaCl, 2 g of NH_4_SO_4_, 1 g of MgSO_4_.7H_2_O, 1 g of K_2_HPO_4_ and 1 l of distilled water; PM2, 10 g of starch, 0.3 g of casein, 2 g of KNO_3_, 4.6 g of NaCl, 2 g of K_2_HPO_4_, 1 0.05 g of MgSO_4_.7H_2_O, 0.02 g of CaCO_3_, 0.01 g of FeSO_4_.7H_2_O, 1 mg of ZnSO_4_.7H_2_O, 18 g of agar and 1 litre of distilled water; and PM3, 10 g of starch, 4 g of yeast extract, 5 g of NaCl, 2 g of NH_4_SO_4_, 2 g of MgSO_4_.7H_2_O, 1 g of K_2_HPO_4_, 1 gm of CaCO_3_, 0.010 g of FeSO_4_.7H_2_O, 0.001 g of ZnSO_4_.7H_2_O, 0.001 g of MnCl_2_.4H_2_O, 0.001 g of CuSO_4_.5H_2_O and 1 l of distilled water. The plates were incubated at 29°C for 10–20 days to attain enough growth over the production media.

For the initial antibacterial screening, agar plugs of 6 mm in diameter were cut from the 10 days old agar plates and plugged into the wells bored using sterile cock borer (diameter of 6 mm) in Mueller Hinton agar plates seeded with different bacteria. The agar plugged plates were incubated at 37°C for 24 h and observed for zone of inhibition around the inserted agar plugs.

For antifungal screening, the actinomycetes were point inoculated on production media at 30 mm distance from the center of plate. Fungal mycelial-disks (6 mm in diameter) prepared from growing margin of cultures of test fungal strains and placed in the center of plate. Antifungal activity was indicated as mycelia growth of fungal isolates was inhibited in the direction of active actinomycete isolates.

### Secondary screening for antimicrobials

Those actinomycetes showed positive antimicrobial activity in the primary screening were subjected to antimicrobial compound production in submerged culture and their antimicrobial proficiency was confirmed in triplicates by disc diffusion method (Bauer et al., 1966).

Spore suspensions of active actinomycetes were prepared in distilled water from cultures grown on ISP-4 medium supplemented with 2% of NaCl and 0.4% yeast extract (w/v). The suspensions were added either to ISP-2 broth or modified ISP4 broth in 250 ml Erlenmeyer flasks at a rate of 10^8^ spores in 50 ml liquid medium incubated on a shaker at 120 rpm at 30°C for 10 to 15 days. From the seed cultures, 25 ml aliquots were transferred to 250 ml production media (PM1, PM2, and PM3) and the flasks were incubated for 10–20 days at 30°C while shaking at 120 rpm. The culture broths were centrifuged at 10,000 rpm for 10 min to separate the mycelial biomass. Ethyl acetate was added to the supernatants in 1:1 proportion and the mixtures were agitated for 45 min. The solvent layers were separated from broths and centrifuged at 5000 rpm for 15 min to remove traces of fermentation broth. The ethyl acetate fractions were evaporated and the resultant crude compounds were resuspended in 50 μl of methanol which were then assayed for antimicrobial activity.

For the evaluation of antimicrobial activity, Mueller Hinton agar plates were inoculated with indicator strains by spreading the microbial inoculums on the surface of the media. The extracts were loaded on 6 mm sterile discs, dried and placed on the surface of the medium inoculated with bacterial test strains and the plates were incubated at 37°C for 24 h. At the end of incubation period, inhibition zones formed around the disc were measured with transparent ruler in millimeter. In the case of antifungal assay the same procedure was practiced with fungal strains on potato dextrose agar instead of Mueller Hinton agar.

### Nucleotide sequence accession numbers

The 16S rRNA gene sequences of representative strains have been deposited in the GenBank database under the accession numbers KC012637- KC012641.

## Results

### Physico-chemical characteristics

Physico-chemical characteristics of a saltern soil sample (Table 1) were comparable with that of other solar salterns previously reported elsewhere (Cai et al., 2009). The soil was alkaline in nature with pH 9.1. Electric conductivity was measured at 14.21 dSm^−1^ with predominance of sodium and chloride. At the time of sample collection, the temperature of the site was measured at 29°C.

**Table 1:** **Physicochemical parameters observed in the soil sample collected from the inland solar saltern**.

**pH**	**EC (dSm-1)**	Values in ppm^*^					
		**Organic C**	**T. Nitrogen**	**T. Sodium**	**T. Potassium**	**Cl**	**Mg**
9.1	14.21	980	43	4240	253	2360	00

* Values are in ppm except pH and EC.

### Selective isolation of actinomycetes

**Figure 2:**
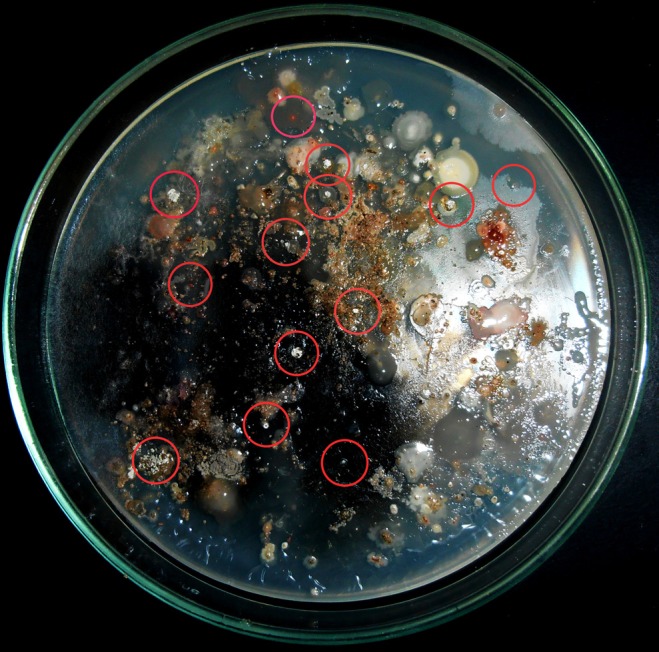
**Stamping method employed for isolation of actinomycetes**. Appearance of actinomycete like colonies encircled on isolation media stamped with ground soil samples.

A total of 14 actinomycete isolates including slow growing and rare actinomycete were isolated from saltpan soil samples using two sample processing methods and three different isolation media with prolonged incubation period. Among the sample-processing methods, stamping facilitated the isolation of 9 (64%) actinomycete strains including members of some rare genera. Appearance of actinomycete like colonies on isolation media stamped with a ground soil sample shown in Figure 2. All the isolates were found to grow slowly on agar media.

### 16S rDNA amplification and ARDRA

**Figure 3:**
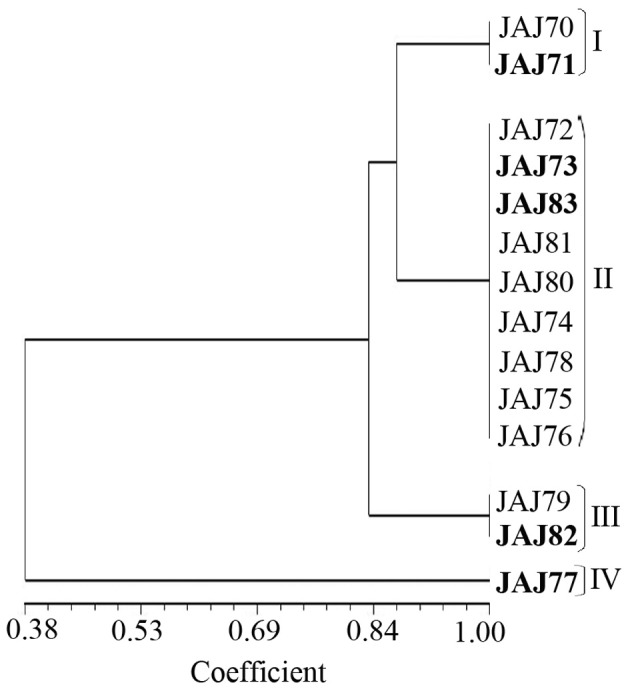
**UPGMA dendrogram shows the clustering of 14 actinomycete isolates generated from amplified ribosomal DNA restriction analysis with restriction endonuclease *Hae*III and *Hinf*I, using the UPGMA algorithm and the Jaccard's coefficient**. The Roman numerals I to IV represent the four clusters obtained in the analysis. The isolates subjected to sequencing analysis are highlighted in boldface.

PCR amplification of 16S rDNA yielded a single amplicon of ~1500 bp for all the isolates. Restriction digestion of these amplicons with *Hae*III and *Hinf*I yielded different profiles characterized by 2–4 fragments ranging from 100 to 800 bp in size for the different isolates. The actinomycete isolates were clustered into four groups in UPGMA dendrogram (Figure 3) inferred from ARDRA patterns obtained with *Hae*III and *Hinf*I. Group II has maximum of 9 isolates, group I and III have 2 isolates each and groups IV has single isolate.

### Actinomycete community composition and phylogenetic analysis

**Figure 4:**
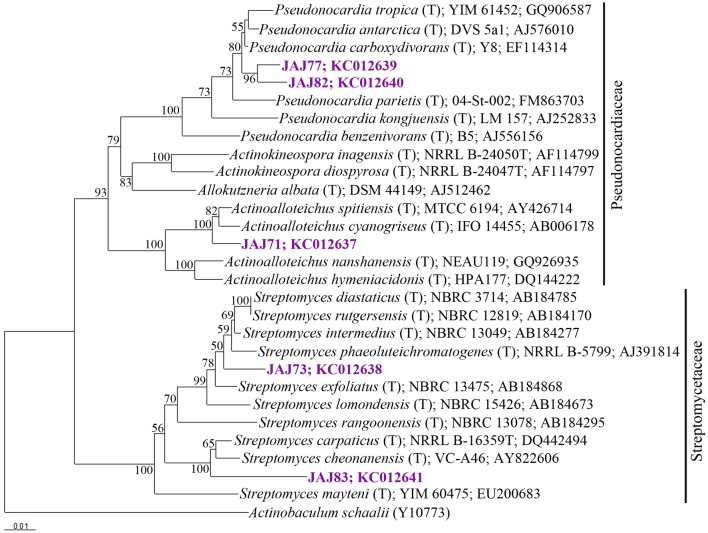
**Neighbor-joining tree based on 16S rRNA gene sequences of selected isolates from inland solar salterns, showing the phylogenetic relationship of isolates and related genera**. Bootstrap values are expressed as percentages of 1000 replications. Bootstrap values, >50% are shown at branch points. *Actinobaculum schaali*^T^ (Y10773) was used as out-group. Score bar represents 1 nucleotide substitution per 100 nucleotides.

16S rDNA of selected five isolates belonging to different clusters established by ARDRA was sequenced and used to determine the diversity among the isolates. The phylogenetic tree (Figure 4) inferred by 16S rDNA sequence indicated that they should be classified in following 3 genera belong to 2 families; Streptomycetaceae and Pseudonocardiaceae.

#### Streptomyces

The most abundant group of isolates was affiliated with the genus *Streptomyces* belong to Streptomycetaceae family, represented by a ribotype comprised 10 isolates accounting for 64% of the total actinomycete population. From these, two isolates were subjected to 16S rDNA based phylogenetic analysis and were found to be affiliated to genus *Streptomyces*. Isolate JAJ73 formed a distinct branch between *Streptomyces phaeoluteichromatogenes* and *Streptomyces exfoliates* in phylogenetic tree (Figure 4) with 98.9 and 98.8% sequence similarity, respectively. Another isolate JAJ83 clustered with *Streptomyces carpaticus* and *Streptomyces cheonanensis* while forming a separate branch. The strain shared 97.6% sequence identity with *Streptomyces carpaticus* and 97.3% with *Streptomyces cheonanensis.*

#### Pseudonocardia

The second most dominant genus among the isolates was *Pseudonocardia*, represented by two different ribotypes accounting for 21% of total actinomycete isolates. Ribotypes JAJ77 and JAJ82 formed distinct cluster within a major cluster comprising closely related members of genus *Pseudonocardia* with 96.8–98.9% of sequence similarity. Nearest type strains were *Pseudonocardia tropica* and *Pseudonocardia antarctica.*

#### Actinoalloteichus

The genus *Actinoalloteichus* was represented by a ribotype comprised of two isolates JAJ70 and JAJ71 (14%). 16S rDNA sequence analysis of JAJ71 revealed their affiliation to *Actinoalloteichus*. In the phylogenetic tree, the isolate JAJ71 was clustered with *Actinoalloteichus spitiensis* and *Actinoalloteichus cyanogriseus* with 99.3 and 99.6% sequence similarity, respectively.

### Morphology of actinomycetes

**Figure 5:**
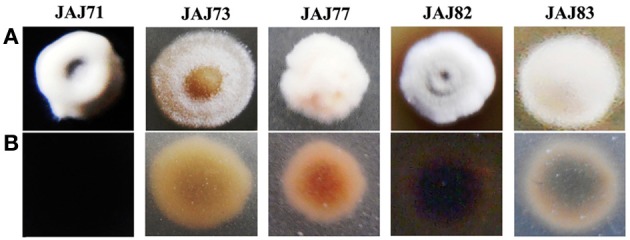
**Colony morphology of representative actinomycete isolates derived from inland solar salterns. (A)** represents the colony view from aerial mycelium side and **(B)** is the substrate mycelium view. Colonies were distinguished based on the color of aerial and substrate mycelia and sporulation.

Morphology of representative actinomycete isolates was visually observed and their aerial and substrate mycelium are shown in Figure 5. All the isolates grew well on IMb with characteristic musty odour, dimorphic mycelium, spore formation and non-motile leathery colonies. *Actinoalloteichus* sp. JAJ71 grew well as white puffy colonies with dark bluish black diffusible pigment, white aerial mycelium and black substrate mycelium. *Streptomyces* sp. JAJ83 produced snow white aerial mycelium and gray substrate mycelium. Whereas, *Streptomyces* sp. JAJ73 produced white aerial mycelium and wheat color substrate mycelium. *Pseudonocardia* sp. JAJ77 produced white aerial mycelium and orange substrate mycelium without diffusible pigment. *Pseudonocardia* sp. JAJ82 grew with brown diffusible pigment, gray aerial mycelium and brown substrate mycelium.

### Tolerance to NaCl

**Figure 6:**
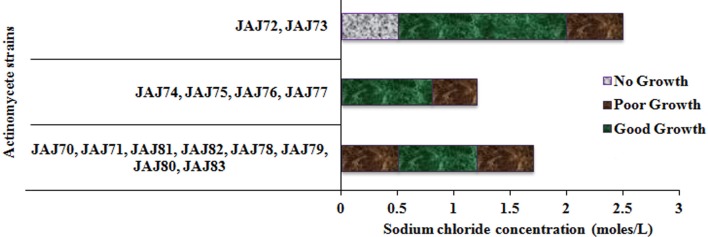
**Tolerance and growth of solar saltern based actinomycetes under different concentration of NaCl**.

The saltern based actinomycetes showed different levels of salt tolerance (Figure 6). Four actinomycete strains (JAJ74, JAJ75, JAJ76, and JAJ77) grew well in the absence of NaCl as well as up to 0.85 M NaCl while grew mildly beyond 0.85 M NaCl up to 1.2 M NaCl in the culture medium. Eight actinomycete strains (JAJ70, JAJ71, JAJ78, JAJ79, JAJ80, JAJ81, JAJ82, and JAJ83) grew optimally in the presence of 0.5–1.2 M and mildly in absence and 1.7 M NaCl. Strains JAJ72 and JAJ73 grew well in the presence of 0.5–2.0 M NaCl and tolerated maximum of 2.5 M NaCl in the culture medium.

### *In vitro* antimicrobial activity

**Figure 7:**
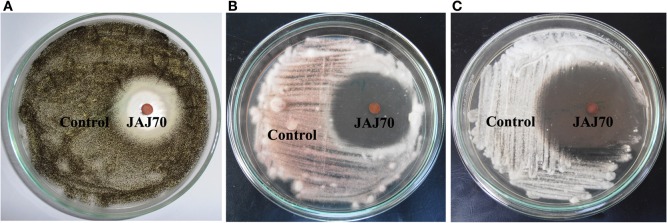
**Antifungal activity by disc diffusion method for *Actinoalloteichus* sp. JAJ70 against three fungal strains (A) *Aspergillus niger*, (B) *Fusarium oxysporum*, and (C) *Alternaria alternata***.

In the primary screening, four actinomycetes (JAJ70, JAJ73, JAJ77, and JAJ82) were found to be active against at least one of the tested bacteria and fungi. Among the four antagonistic actinomycetes, JAJ73 and JAJ82 showed antifungal activity, JAJ77 showed antibacterial activity and JAJ70 showed both the antifungal and antibacterial activity. The actinomycetes showed antimicrobial activity in primary screening were further recognized to produce antimicrobial compound in the batch of submerged fermentation process with three different production media (Tables 2, 3). All the three production media, PM1, PM2, and PM3 supported antimicrobial compound production in all the antagonistic actinomycetes, however the measure of antimicrobial compound varied from one medium to another (Tables 2, 3). PM3 favored higher antimicrobial compound production in the all the actinomycetes. *Actinoalloteichus* sp. JAJ70 exhibited highest antifungal activity (up to 44 ± 1.2 mm) followed by *Streptomyces* sp. JAJ73 (14 ± 1.20 mm) and *Pseudonocardia* sp. JAJ82 (16 ± 1.52 mm) against the fungal strains. Furthermore, *Actinoalloteichus* sp. JAJ70 exhibited considerable antibacterial activity (up to 35 ± 0.88 mm) followed by *Pseudonocardia* sp. JAJ77 with up to 21 ± 1.15 mm diameter of zone of inhibition against the bacterial strains. Figure 7 shows antifungal activity by disc diffusion method for *Actinoalloteichus* sp. JAJ70 against three fungal strains.

**Table 2:** **Antibacterial activity of saltern actinomycetes against five test bacteria**.

**Isolates**	**Medium**	**Antimicrobial activity/Zone of Inhibition (mm ± SEM)**				
		***B. subtilis***	***P. vulgaris***	***K. pneumoniae***	***S. aureus***	***S. typhi***
*Actinoalloteichus* sp. JAJ70	PM1	19 ± 1.15	20 ± 1.45	19 ± 0.88	29 ± 1.73	18 ± 0.66
	PM2	15 ± 1.20	17 ± 1.20	19 ± 1.45	27 ± 0.66	13 ± 0.88
	PM3	24 ± 1.15	25 ± 0.57	25 ± 0.57	35 ± 0.88	21 ± 1.20
*Pseudonocardia* sp. JAJ77	PM1	14 ± 1.20	17 ± 1.52	13 ± 1.45	21 ± 1.15	13 ± 0.33
	PM2	11 ± 0.88	15 ± 1.15	12 ± 0.88	16 ± 0.33	12 ± 1.00
	PM3	11 ± 0.88	12 ± 0.57	11 ± 1.00	15 ± 0.57	11 ± 0.66

**Table 3:** **Antifungal activity of saltpond actinomycete isolates against three test fungi**.

**Isolates**	**Medium**	**Antimicrobial activity/Zone of Inhibition (mm)**		
		***A. niger***	***F. oxysporum***	***A. alternata***
*Actinoalloteichus* sp. JAJ70	PM1	14 ± 1.15	25 ± 0.88	30 ± 1.20
	PM2	13 ± 0.33	13 ± 1.20	16 ± 0.88
	PM3	19 ± 1.73	33 ± 1.76	44 ± 1.20
*Streptomyces* sp. JAJ73	PM1	11 ± 0.33	13 ± 1.15	10 ± 0.88
	PM2	12 ± 1.76	14 ± 1.20	11 ± 1.30
	PM3	10 ± 1.00	11 ± 1.52	10 ± 0.57
*Pseudonocardia* sp. JAJ82	PM1	14 ± 1.15	16 ± 1.52	12 ± 1.45
	PM2	12 ± 1.20	12 ± 0.57	10 ± 0.57
	PM3	15 ± 1.15	14 ± 1.45	11 ± 0.88

## Discussion

The present study offers the first overview on molecular phylogeny, salt-tolerance and antimicrobial activity of slow growing and rare actinomycetes isolated from the inland solar salterns located in the vicinity of Sambhar Salt Lake, India. Physicochemical characteristics from this study confirm that this environment is a hypersaline zone. Similar hypersaline environments have recurrently been explored as source of actinomycetes with biotechnologically important secondary metabolites and enzymes (Thumar et al., 2010; Hamedi et al., 2013). Hence, disclosing the molecular phylogeny of actinomycetes inhabiting such hypersaline environments is highly desirable. In the present work, the selective isolation methods employed stamping, heat and dilution were used to acquire actinomycetes and succeeded with isolation of both *Streptomyces* (common actinomycete) and non-*Streptomyces* (rare actinomycetes) such as *Actinoalloteichus* and *Pseudonocardia*. Similarly, selective sample processing methods and isolation media have been used to isolate taxonomically diverse actinomycetes from saline environments (Jensen et al., 2005). The successful isolation of most of the isolates using stamping method was in line with previously reported employability of this method for isolation of actinomycetes from saline environments (Gontang et al., 2007; Jose and Jebakumar, 2012).

Phylogenetic affiliation of the isolated actinomycetes was studied based on the highly recognized eubacterial phylogenetic marker, 16S rRNA gene (Ludwig and Schleifer, 1994). ARDRA was employed with four-cutter restriction enzymes *Hae*III and *Hinf*I to assist in distinguishing among the taxonomic groups. The ARDRA has previously been used to discriminate the microbes at inter genus and intra genus levels (Wilson et al., 1998; Cook and Meyers, 2003; Ahmad et al., 2011). In the present study, five actinomycete isolates were selected as representative strains from the entire actinomycete population using the ARDRA. Phylogenetic analysis based on 16S rRNA gene sequences of representative isolates suggested that the obtained actinomycete population is composed of *Streptomyces, Pseudonocardia* and *Actinoalloteichus*. Of these, *Streptomyces* was found to be predominant genus among the actinomycetes isolated from the inland solar salterns. The predominance of the *Streptomyces* has also been observed in other saline environments (Kushner, 1978; Satheeja and Jebakumar, 2011). Following the *Streptomyces*, rare genera *Pseudonocardia* and *Actinoalloteichus* were allocated to second position in predominance among the actinomycete isolates. Members affiliated with genus *Pseudonocardia* have been reported from marine hypersaline environments (Peela et al., 2005; Tian et al., 2013). In contrast, in the present study, isolates affiliated with *Pseudonocardia* were isolated from inland hypersaline environment. *Actinoalloteichus* was another rare genus isolated from the inland solar salterns. Currently, this genus comprises four validly described members which include *Actinoalloteichus cyanogriseus* isolated from a soil sample collected from the Yunnan province of China (Liu et al., 2006), *Actinoalloteichus spitiensis* isolated from cold desert of the Indian Himalayas (Tamura et al., 2000), *Actinoalloteichus hymeniacidonis* isolated from marine sponge (Singla et al., 2005) and *Actinoalloteichus nanshanensis* isolated from the rhizosphere of a fig tree (Zhang et al., 2006). In the present study, existence of members affiliated with *Actinoalloteichus* has been found first time in inland solar salterns associated with a salt Lake. The scrutiny of the 16S rDNA phylogenetic tree suggests that most of the isolates may represent novel species. Further polyphasic taxonomic studies are needed to describe the novel species.

Salt tolerance analysis revealed that most of the isolated actinomycetes grew more rapidly at salt concentration between 0.5 and 2.0 M. Halophilic micro-organisms are categorized as extreme halophiles (growing best in media containing 2.5–5.2 M salt), borderline extreme halophiles (growing best in media containing 1.5–4.0 M salt) and moderate halophiles (growing best in media containing 0.5–2.5 M salt) (Kushner, 1978). According to this scheme, two isolates JAJ72 and JAJ73 could be categorized under moderate halophiles as they grow best in 0.5–1.7 M salt. Halotolerants are those do not show an absolute requirement to salt for growth but grow well up to often very high salt concentrations (Xiang et al., 2011). In the current actinomycete community, 12 isolates could be categorized under this halotolerant group as they grow in the absence of NaCl as well tolerate higher concentration of NaCl up to 1.7 M. Similar halotolerance and halophilism has also been observed in actinomycetes isolated from other salt lake environments (Oren, 2002; Cai et al., 2009).

To evaluate the potential of saltern based actinomycetes for antibiotic discovery programs, they were screened for their antimicrobial activity against a range of microorganisms. Antimicrobial activity was observed in both slow growing and rare genera of actinomycetes. Among them, members of rare genera *Pseudonocardia* and *Actinoalloteichus* were found to be more active against tested bacteria and fungi. The strong inhibitory activities of these rare actinomycetes were in accordance with recently reviewed biotechnological importance of halophilic and halo-tolerant rare actinomycetes (Tiwari and Gupta, 2012; Hamedi et al., 2013) and suggested that these actinomycetes may be potential candidates for the production of antimicrobial compounds. Some of the strains are presently being studied to identify the nature of antimicrobial compounds and to assess their novelty.

Concisely, our successive efforts to isolate novel actinomyctes from coastal (Jose and Jebakumar, 2012) and inland (this study) solar salterns revealed the inhabitance of diverse actinomycetes in hypersaline Indian solar salterns. This study contributes to our acquaintance of solar saltern-associated actinobacteria and further augments the array of actinomycetes available for antibiotic discovery programs.

### Conflict of interest statement

The authors declare that the research was conducted in the absence of any commercial or financial relationships that could be construed as a potential conflict of interest.

## Abbreviations

PCR: polymerase chain reaction
ARDRA: amplified ribosomal DNA restriction analysis
16S rDNA: 16S ribosomal DNA.
